# No impairment of quality of life after radiotherapy for prostate cancer

**DOI:** 10.1038/s41598-024-84257-8

**Published:** 2024-12-31

**Authors:** Maria M. Meier, Oliver Koelbl, Isabella Gruber

**Affiliations:** 1 University of Regensburg, Universitätsstraße 31, Regensburg, Germany; 2 Department of Radiation Oncology, University Hospital of Regensburg, Franz-Josef-Strauß Allee 11, Regensburg, Germany

**Keywords:** Prostate cancer, Health-related quality of life, Radiotherapy, Radiation therapy, Patient-reported outcome, Quality of life, Prostate cancer

## Abstract

There are concerns that radiotherapy for prostate cancer influences health-related quality of life in the long term. Furthermore, it is unclear whether postoperative radiotherapy is associated with a different quality of life due to a higher treatment burden compared to patients having received definitive radiotherapy for prostate cancer. This study enrolled 247 patients with localized or locally advanced prostate cancer who received external radiotherapy between 2011 and 2021. Health-related quality of life was assessed at a median of 63.6 months after radiotherapy using the European Organization for Research and Treatment of Cancer quality of life questionnaire (EORTC QLQ-C30) with 145 patients returning questionnaires (response rate, 58.7%). Four patients treated with adjuvant radiotherapy were excluded due to the small number, resulting in 141 participants who received salvage radiotherapy (70 men) or definitive radiotherapy (71 men). The study compared the quality of life with age- and sex-matched German normative data. Patients completed the questionnaires after a median time of 60.3 and 65.2 months after salvage and definitive radiotherapy. The median patient age was higher in the definitive than in the salvage radiotherapy group (at radiotherapy, 72 vs. 69 years; at the survey, 79 vs. 75 years). Global health status, functional scales (physical, role, emotional, cognitive, and social), and symptom scales were not different between cancer patients of the same age group treated with salvage and definitive radiotherapy. The comparison with age- and sex-matched normative data revealed that salvage and definitive radiotherapy did not impair the global health status in patients of any age group. Physical functioning in patients < 70 years was significantly better in salvage and definitive radiotherapy groups compared to normative data while showing clinical relevance. Yet, social functioning was significantly lower in patients ≥ 70 years of the salvage radiotherapy group compared to normative data, while this difference lacked clinical significance. Regardless of the type of radiotherapy applied, cancer patients had no statistically or clinically relevant higher symptom burden compared to normative data. Quality of life was not clinically relevant influenced by radiotherapy, regardless of whether patients received salvage or definitive radiotherapy. Yet, longitudinal measurements of quality of life after radiotherapy are required to detect fluctuations in quality of life.

## Introduction

Prostate cancer is the most commonly diagnosed cancer among men in Germany. Definitive radiotherapy and radical prostatectomy are both treatment options associated with different side effects influencing patients´ quality of life^1,2^. Health-related quality of life after treatment is of high interest as patients with prostate cancer have an extended life expectancy^1,3^. The expected side effects and quality of life after treatment are relevant information for patients before a decision for therapy can be made. Although advancements in radiotherapy techniques (intensity-modulated radiotherapy, IMRT, and volumetric modulated arc therapy, VMAT) reduced treatment-related side effects^4^, radiotherapy for prostate cancer treatment is critically and ongoing debated^5^. Early randomized and controlled studies used older irradiation techniques, resulting in relatively high rates of late effects after radiotherapy^6,7^. However, modern radiotherapy techniques proved comparatively low toxicity^8^. The primary aim of this study was to analyze the health-related quality of life of long-term survivors of prostate cancer after modern radiotherapy by using the European Organization for Research and Treatment of Cancer quality of life questionnaire (EORTC QLQ-C30). Comparisons with sex- and age-matched normative data^9^ should simplify the interpretation of the results and clarify whether radiotherapy influences the quality of life in the long term. Furthermore, the data should provide information on whether patients receiving postoperative radiotherapy dealing with additional side effects from surgery have a different health-related quality of life in the long term compared to patients receiving definitive radiotherapy.

## Methods

### Patients and data collection

**Fig. 1:**
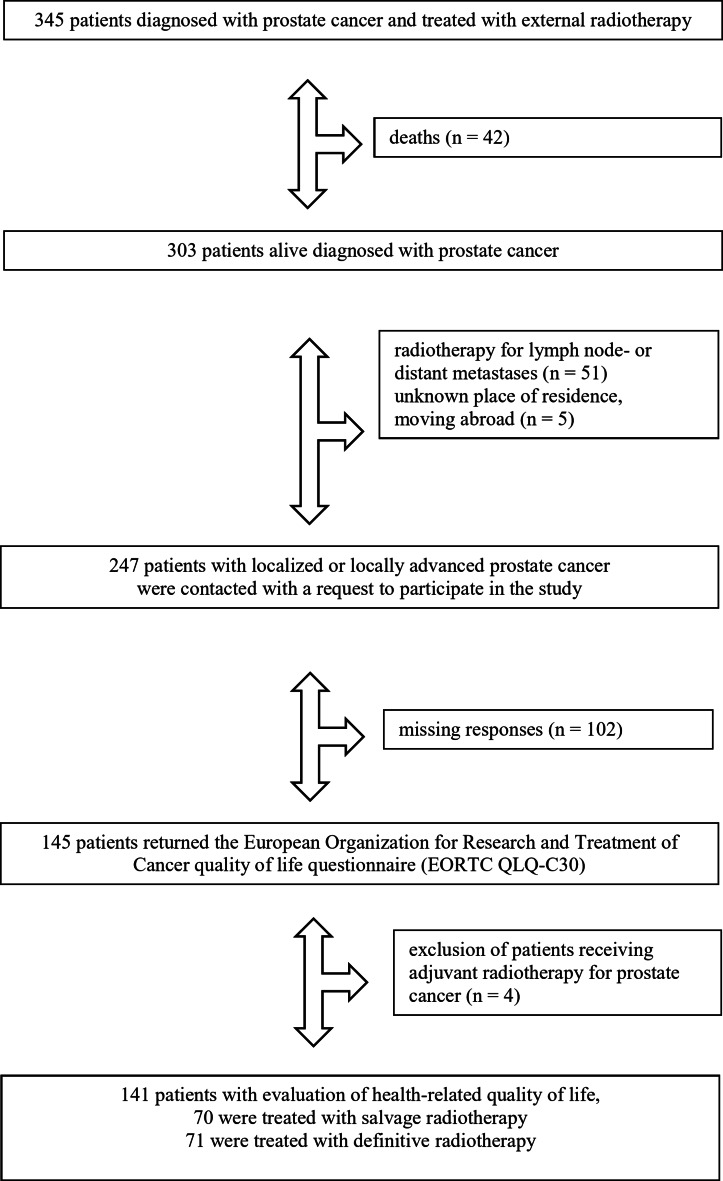
Flow chart.

From January 2011 until December 2021, 345 patients diagnosed with prostate cancer were treated with external radiotherapy at the Department of Radiation Oncology of the University Hospital Regensburg (Fig. 1). Forty-two patients (12.2%) died during follow-up, while 303 patients (87.8%) were alive. Patients who received radiotherapy for lymph node- or distant metastases were excluded (*n* = 51). The current place of residence was known for 247 patients. These patients were asked in writing to participate in the study. One hundred forty-fife patients returned the European Organization for Research and Treatment of Cancer quality of life questionnaire (EORTC QLQ-C30, *n* = 145, response rate, 58.7%). The study excluded four patients treated with adjuvant radiotherapy due to the small number of patients. In summary, the study included 141 participants, of whom 70 had received salvage and 71 definitive radiotherapy.

### Cancer characteristics

The tumor stages (localized vs. locally advanced) at the time of diagnosis of prostate cancer were similar between patients receiving salvage (localized, *n* = 18; locally advanced, *n* = 52) and definitive radiotherapy (localized, *n* = 28; locally advanced, *n* = 43; *P* = 0.106). The distribution of Gleason scores was not different between groups (*P* = 0.191). Most patients had a Gleason score of 7a at the time of diagnosis of prostate cancer (salvage radiotherapy vs. definitive radiotherapy; *n* = 20 vs. *n* = 17), a Gleason score of 7b (*n* = 18 vs. *n* = 11) or a Gleason score of 6 (*n* = 10 vs. *n* = 17). Gleason scores of 8 (*n* = 8 vs. *n* = 14) and 9 (*n* = 13 vs. *n* = 9) were less common. Patients receiving salvage radiotherapy had a median PSA of 0.3 ng/ml (IQR, 0.2–0.7) before radiotherapy. The median PSA before definitive radiotherapy was 7.9 ng/ml (IQR, 4.8–13.2).

### Radiotherapy

All patients received instructions to constantly fill the bladder and empty the rectum before CT planning and daily radiotherapy. Patients were immobilized with a blue bag vacuum cushion for precise and reproducible molds of the patient´s contour and received a planning computer tomography. We administered no hydrogel spacers. Median doses applied with salvage and definitive radiotherapy were 70 Gy (IQR, 70.0–70.0) and 78.0 Gy (IQR, 77.9–78.0), respectively. Doses were given to the planning target volume (PTV) comprising the region of the prostate and seminal vesicles followed by a boost. Organs at risk included the bladder, rectum, anal canal, bowel bag, and femoral heads. Patients received five fractions/week using linear accelerators. Radiotherapy was performed using intensity modulated radiation therapy (IMRT) and volumetric modulated arc therapy (VMAT) with 6 MV photons. Treatment positions were regularly monitored using kV imaging.

### Definitions of adjuvant and salvage radiotherapy

Adjuvant radiotherapy refers to radiotherapy after radical prostatectomy after the defined PSA zero range has been reached (PSA < 0.1 ng/ml). Patients who did not have a PSA < 0.1 ng/ml after radical prostatectomy (persistent PSA) or patients with rising PSA were given salvage radiotherapy.

### Questionnaire

EORTC QLQ-C30 (European Organization for Research and Treatment of Cancer Quality of Life Questionnaire Core 30) version 3.0 is a questionnaire that analyzes the health-related quality of life of cancer patients in general^10^. The EORTC QLQ-C30 comprises 30 questions with five functional scales (physical, role, emotional, cognitive, and social functioning), nine symptom scales (fatigue, nausea and vomiting, pain, dyspnea, insomnia, appetite loss, constipation, diarrhea, and financial difficulties), and global health status. For EORTC QLQ-C30, the average of the corresponding items is calculated (raw score). Finally, the raw scores are linearly transformed to a 0-100 range. A higher score on the functional scales and the global health status indicates a higher functioning and quality of life. A higher symptom scale is associated with a greater severity of problems. The study compared the results of the EORTC QLQ-C30 with up-to-date sex- and age-matched German normative data^9^. We separated all patients into groups depending on their age at the survey (≤ 69.9 years, *n* = 67 vs. ≥ 70.0 years, *n* = 74) for comparisons with age- and sex-matched normative data. If at least half of the items from a scale were answered, the study used all the items and applied the standard equation as advised in the EORTC QLQ-C30 scoring manual^11^, ignoring any items with missing values. If not at least half of the items from a scale have been answered, the scale score was set to missing. In summary, four scales were set to missing. The local Ethics Board of the University of Regensburg approved this study (approval´s number, 23-3287-101, date 29 March 2023). All patients gave written informed consent.

### Statistical analysis

Demographic and tumor characteristics were presented as mean, standard deviation (SD), median, and interquartile range (IQR). The Mann-Whitney U test was used to compare quality of life measures between groups. Categorical variables were analyzed with chi-square tests. We compared the quality of life scores of the patients and age- and sex-adjusted normative data with one sample t-tests. The study tested for statistical differences and clinical relevance between patients and normative data. The study set a difference of 0.5 of the standard deviation to determine the clinical relevance of patient-reported outcome measures. All tests were performed with a 2-sided *P*-value < 0.05 for statistical significance. Statistical analysis was performed with SPSS 26.0 (SPSS Inc., Chicago, IL, USA) and graphics with Excel (2013, Microsoft Office).

## Results

### Patient characteristics

Seventy patients (*n* = 70) received salvage radiotherapy for prostate cancer, while 71 patients received definitive radiotherapy. The median follow-up from the last day of radiotherapy to the return of questionnaires was 63.6 months (IQR, 34.7–92.7). The median follow-up was not different between the salvage radiotherapy group (60.3 months, IQR, 33.2–95.1) and definitive radiotherapy group (65.2 months, IQR, 37.4–89.1; *P* = 0.743). Patients receiving definitive radiotherapy were older than patients receiving salvage radiotherapy at the time of radiotherapy (72 years, IQR, 66–77 vs. 69 years, IQR, 63–72; *P* = 0.007) and at the time of survey (79 years, IQR, 72–83 vs. 75 years, IQR, 70–80; *P* = 0.009). The median Karnofsky performance status was not different between groups (definitive, 90, IQR, 80–90; salvage, 90, IQR, 80–90) at the time of radiotherapy (*P* = 0.921).

### Health-related quality of life

**Fig. 2:**
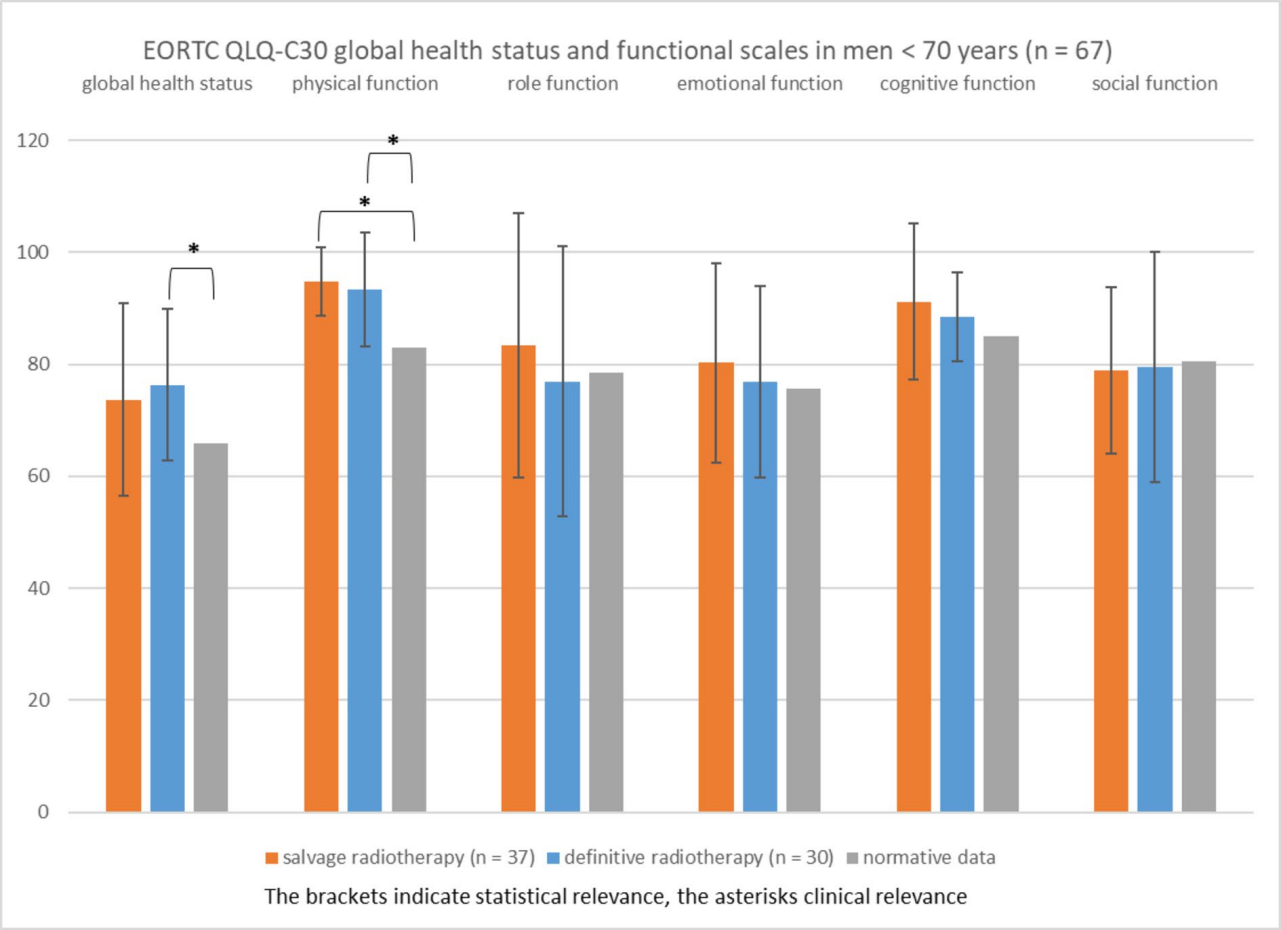
EORTC QLQ-C30 global health status and functional scales in men < 70 years (mean, standard deviation) treated with radiotherapy for prostate cancer compared to age-matched normative data (range 0, most affected to 100, least affected).

Figure 2 shows the EORTC QLQ-C30 global health status and functional scales in cancer patients < 70 years (*n* = 67) at the time of the survey compared to age- and sex-matched normative data for Germany^9^. Cancer patients < 70 years treated with salvage (*n* = 37) and definitive radiotherapy (*n* = 30) did not show different global health status and functional scales. Salvage and definitive radiotherapy were not associated with a lower global health status in patients < 70 years compared to normative data of the same age group. On the contrary, the global health status of patients < 70 years treated with definitive radiotherapy was significantly and clinically relevant higher than the global health status of the age-matched normative data (mean, 76.3 vs. 65.9; *P* = 0.017). Patients treated with salvage radiotherapy (mean, 94.7; *P* < 0.001) and definitive radiotherapy (mean, 93.3; *P* = 0.003) had significantly and clinically relevant better physical functioning compared to normative data (mean, 83.0). Role, emotional, cognitive, and social functions were not lower in patients of both radiotherapy groups compared to normative data of the same age group.

**Fig. 3:**
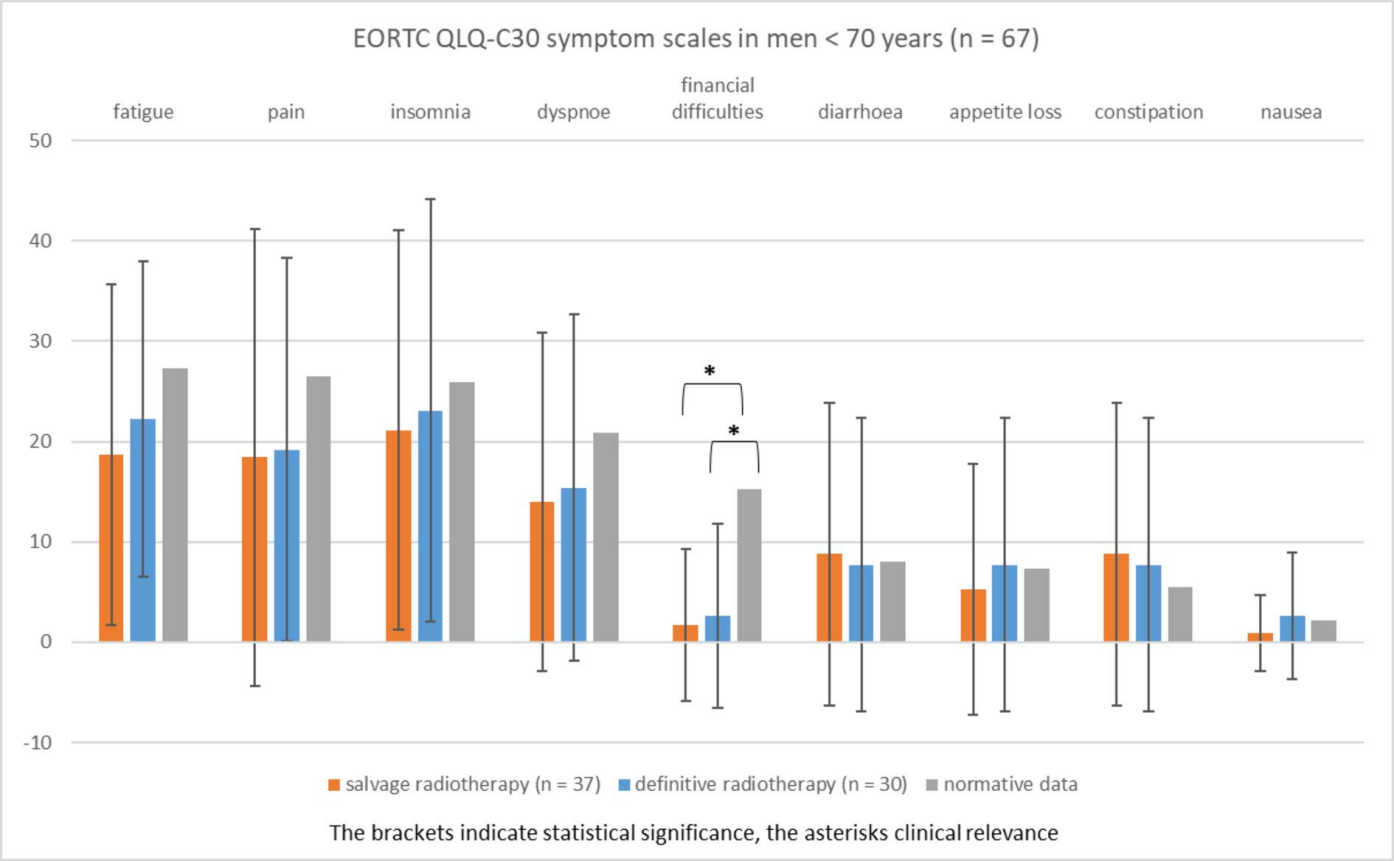
EORTC QLQ-C30 symptom scales in men < 70 years (mean, standard deviation) treated with radiotherapy for prostate cancer compared to age-matched normative data (range 0, least affected to 100, most affected).

Figure 3 illustrates the EORTC QLQ-C30 symptom scales in cancer patients < 70 years (*n* = 67) compared to age- and sex-adjusted normative data^9^. Symptom scales were not different in patients < 70 years treated with salvage (*n* = 37) and definitive radiotherapy (*n* = 30) for prostate cancer.

Regardless of the type of radiotherapy applied, no higher symptom burden was observed in cancer patients < 70 years in comparison to normative data of the same age group. Financial difficulties were significantly and clinically relevant lower in patients treated with salvage radiotherapy (mean, 1.7; *P* < 0.001) and definitive radiotherapy (mean, 2.6; *P* < 0.001) compared to normative data (mean, 15.3).

**Fig. 4:**
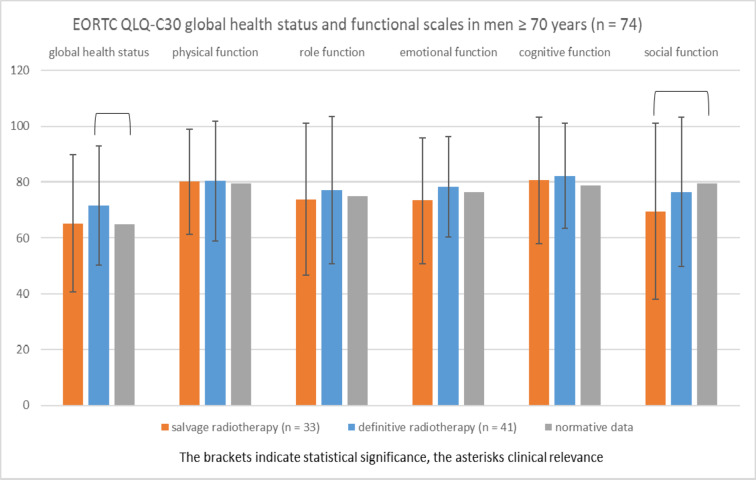
EORTC QLQ-C30 global health status and functional scales in men ≥ 70 years (mean, standard deviation) treated with radiotherapy for prostate cancer compared to age-matched normative data (range 0, most affected to 100, least affected).

Figure 4 shows the EORTC QLQ-C30 global health status and functional scales in cancer patients ≥ 70 years (*n* = 74) compared to age- and sex-adjusted normative data^9^. Cancer patients ≥ 70 years of different treatment groups (salvage [*n* = 33] vs. definitive radiotherapy [*n* = 41]) showed similar global health status and similar functional scales.

The comparison to age-matched normative data revealed that the global health status of patients ≥ 70 years treated with salvage or definitive radiotherapy was not reduced compared to normative data of the same age group. Patients of the definitive radiotherapy group had a significantly better global quality of life compared to normative data of the same age group (71.5 vs. 65.0; *P* = 0.026). However, this difference lacked clinical relevance. Social functioning was significantly lower in patients of the salvage radiotherapy group compared to normative data (69.6 vs. 79.7; *P* = 0.025). However, this difference lacked clinical relevance. Physical, role, emotional, and cognitive functions were not different between cancer patients ≥ 70 years and normative data of the same age group.

**Fig. 5:**
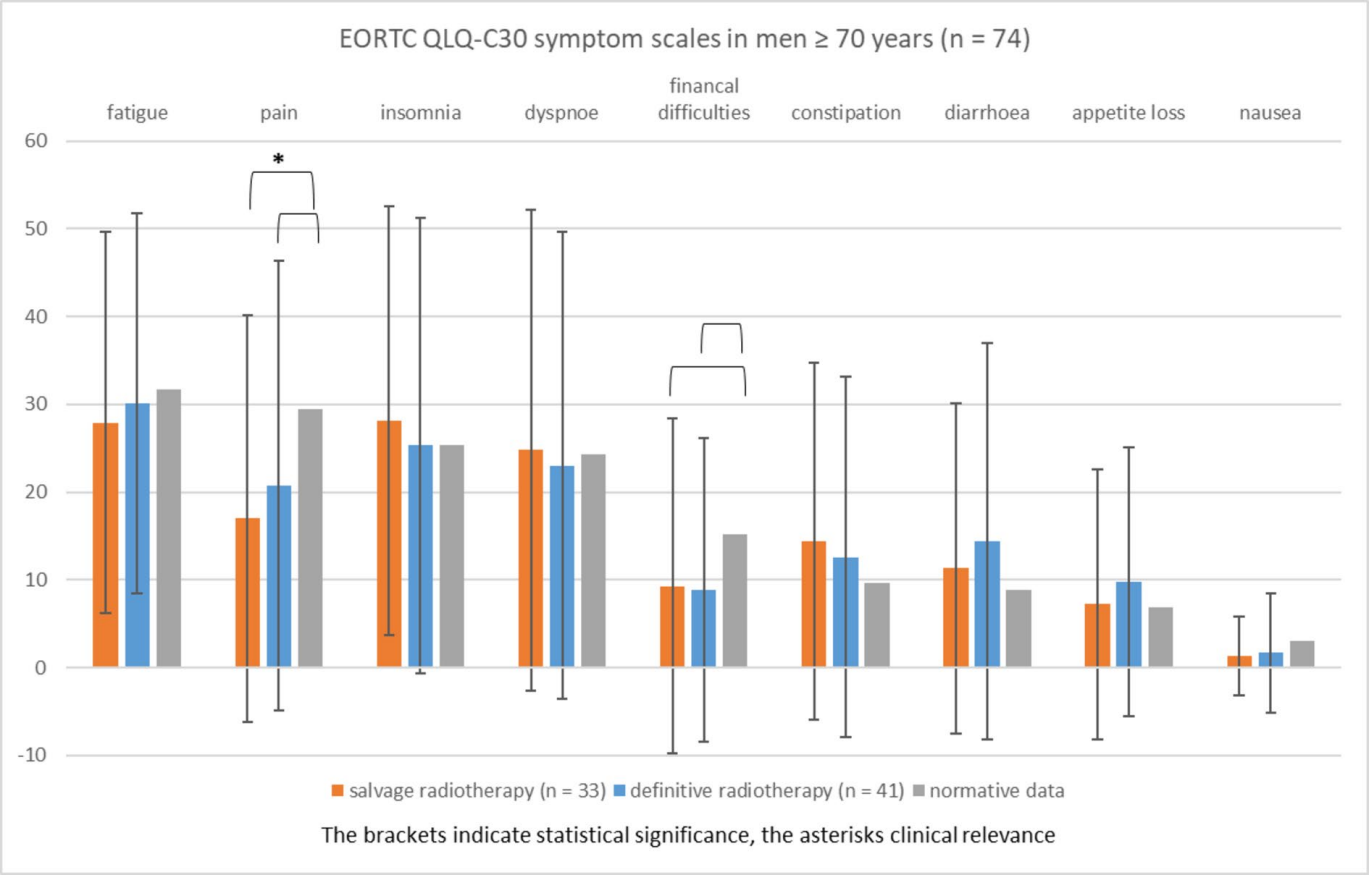
EORTC QLQ-C30 symptom scales in men ≥ 70 years (mean, standard deviation) treated with radiotherapy for prostate cancer compared to age-matched normative data (range 0, least affected to 100, most affected).

Figure 5 shows the EORTC QLQ-C30 symptom scales of cancer patients ≥ 70 years (*n* = 74) compared to normative data of the same age group^9^. EORTC QLQ-C30 symptom scores of patients of both treatment groups (salvage [*n* = 33] vs. definitive radiotherapy [*n* = 41]) were not different.

The comparison to normative data of the same age group revealed that the symptom burden was not higher in patients of any treatment group. Patients treated with salvage radiotherapy had statistically significantly and clinically relevant lower pain scores than men of the age-matched normative data (mean, 17.0 vs. 29.5; *P* < 0.001). The significant difference in pain score (*P* = 0.011) between patients ≥ 70 years treated with definitive radiotherapy (mean, 20.7) and normative data (mean, 29.5) was not clinically relevant. Cancer patients ≥ 70 years treated with salvage radiotherapy (9.3 vs. 15.2; *P* = 0.035) and definitive radiotherapy (8.8 vs. 15.2; *P* = 0.007) had statistically significant lower financial difficulties than men of normative data (mean, 15.2). However, these differences lacked clinical relevance (Fig. 5).

## Discussion

Health-related quality after radiotherapy is of intense research, while patient-reported outcome measures in the long term are of particular interest, as acute toxicity usually resolves quickly. The present study, therefore, analyzed whether external radiotherapy affects the general quality of life in the long term and focused on patients receiving radiotherapy for localized or locally advanced prostate cancer. The median follow-up between radiotherapy and the analysis of quality of life was 5.3 years. This time indicates that health-related quality of life is not affected in the long-term after radiotherapy for prostate cancer. The present study revealed that functional scales were not different between treatment groups (salvage vs. definitive radiotherapy). The global health status of patients of any treatment group was similar or even higher than those of sex- and age-matched normative data, which indicates that radiotherapy is not detrimental in the long term. Symptom scales did not differ between treatment groups (salvage vs. definitive radiotherapy), and symptom burden was the same or even lower than normative data of the same age group. Therefore, patients maintained a good health-related quality of life after radiotherapy for prostate cancer, irrespective of treatment intent. Longitudinal studies applying intensity modulated radiotherapy for prostate cancer support the fact that a temporary worsening of quality of life measures resolves with increasing follow-up after radiotherapy for prostate cancer^12^. The ProtecT (Prostate Testing for Cancer and Treatment) trial compared EORTC QLQ-C30 measures of patients after radiotherapy (74 Gy), prostatectomy, and active monitoring with a follow-up from 7 to 12 years^2^. The study revealed no differences in physical health, mental health, and general quality of life scores at 5 and 10 years^2^. The present study lacks a comparison arm of patients who were treated with radical prostatectomy only making comparisons to other studies difficult. Furthermore, some studies included hypo-fractionated radiotherapy, brachytherapy, or 3D-conformal radiotherapy, which contributed to different results.

The results of the present study are in line with a large study focusing on quality of life in cancer survivors using the EORTC QLQ-C30 questionnaire^3^. Arndt et al.^3^ analyzed the quality of life in long-term cancer survivors 5–16 years after diagnosis of breast, colorectal and prostate cancer in Germany, concluding that the overall quality of life in long-term cancer survivors was comparable to population norms.

The present study has several limitations, including a small study population, which suggests a potential selection bias. Another limitation is the imbalance in patient characteristics. Patients receiving definitive radiotherapy were older both at the time of treatment and at the time of the survey. This age difference introduces the possibility that age-related health declines may confound quality of life outcomes between the two groups. To address this issue, future studies should account for age-related factors, such as baseline comorbidities, to ensure that observed differences in quality of life are attributed to the treatment type rather than age-related health variations. Additionally, relapses following radiotherapy could not be evaluated due to missing PSA data for some patients. Consequently, no conclusions can be drawn regarding whether recurrence status impacts quality of life. Nevertheless, unlike other studies that utilize older radiotherapy techniques, the present study employed modern techniques, ensuring its findings reflect current treatment standards.

## Conclusions

Patients maintained a good long-term quality of life after radiotherapy for prostate cancer, irrespective of treatment intent. The study revealed no clinically relevant impairment in health-related quality of life after salvage and definitive radiotherapy for prostate cancer. Although definitive radiotherapy is associated with less cumulative treatment burden, health-related quality of life was not different between treatment groups. Yet, longitudinal measurements of quality of life after radiotherapy may be more appropriate to detect fluctuations in quality of life.

## Data Availability

The data that support the findings of this study are available on request from the corresponding author. The data are not publicly available due to privacy or ethical restrictions.
